# The effects of oral vitamin D supplementation on the prevention of peritoneal dialysis-related peritonitis: study protocol for a randomized controlled clinical trial

**DOI:** 10.1186/s13063-019-3784-7

**Published:** 2019-11-28

**Authors:** Yu-hui Zhang, Xiao Xu, Hai-chen Pi, Zhi-kai Yang, David W. Johnson, Jie Dong

**Affiliations:** 1 Renal Division, Department of Medicine, Peking University First Hospital, Beijing, China; 2 Institute of Nephrology, Peking University, Beijing, China; 3 Key Laboratory of Renal Disease, Key Laboratory of Renal Disease, Ministry of Health, Beijing, China; 4 Key Laboratory of Renal Disease, Ministry of Education, Beijing, China; 5 Department of Emergency Medicine, Peking University First Hospital, Beijing, China; 6 Department of Nephrology, Princess Alexandra Hospital, Brisbane, Australia; 7 Australasian Kidney Trials Network, University of Queensland, Brisbane, Australia; 8 Translational Research Institute, Brisbane, Australia

**Keywords:** Natural vitamin D3, Peritoneal dialysis, Peritonitis, Serum 25-hydroxyvitamin D, Randomized controlled trial

## Abstract

**Background:**

Vitamin D deficiency has been shown to be closely associated with peritoneal dialysis (PD)-related peritonitis. The aim of this study is to examine the feasibility of conducting a large, powered randomized controlled trial to determine the effects of vitamin D supplementation on the risk of PD-related peritonitis in patients who have already experienced an episode of peritonitis.

**Methods:**

This prospective, open-label randomized controlled pilot trial with blinded end-points aims to determine the feasibility of oral vitamin D supplementation and to explore its effects on the risk of subsequent PD-related peritonitis among PD patients who have recovered from a recent episode of peritonitis. Eligible patients will be randomized 1:1 to either oral vitamin D supplementation (2000 IU per day; intervention group) or no vitamin D supplementation (control group) in addition to usual care according to International Society for Peritoneal Dialysis guidelines. The sample size will be 30 patients for both groups. All participants will be followed for 12 months. The primary outcome is the assessment of feasibility (recruitment success, retention, adherence, safety) and fidelity (change in serum 25-hydroxyvitamin D level during follow-up) for a large, powered randomized controlled trial to determine the effects of vitamin D on the risk of PD-related peritonitis in the future. Secondary outcomes include time to peritonitis occurrence, recovery of peritonitis, peritonitis-related transition to hemodialysis, and peritonitis-related death (defined as death within 30 days of peritonitis onset).

**Discussion:**

This is the first randomized controlled trail investigating the effects of vitamin D supplementation on the risk of subsequent PD-related peritonitis among patients on PD. The findings for this pilot study will determine the feasibility of conducting a full-scale randomized controlled trail, which may provide a new strategy for preventing PD-related peritonitis among PD patients.

**Trial registration:**

Clinicaltrails.gov, NCT03264625. Registered on 29 August 2017.

## Background

Peritoneal dialysis (PD)-related peritonitis is the most common infectious complication for patients on PD, and is a major contributor to treatment failure, hospitalization, and death [1]. Indeed, PD-related infection is considered the top, most critical research priority in PD by patients, caregivers, and clinicians [2, 3]. Almost 40% of patients will experience one or more episodes of peritonitis during extended follow-up on PD [4]. Furthermore, once patients experience an initial peritonitis episode, they are at increased risk of a subsequent, more serious peritonitis event after a median time interval of 8 months [5, 6]. Key risk factors for the development of peritonitis include innate and adaptive immune dysfunction, inflammation, and malnutrition [7–10].

Vitamin D deficiency is commonly associated with malnutrition [6] and is observed in the majority of dialysis patients [11, 12]. In addition to playing a role in the regulation of both the innate and adaptive immune systems [13, 14], vitamin D deficiency has been found to be independently predictive of increased risks of a variety of infectious diseases in the general population [15–17]. Previous observational cohort studies have also reported that vitamin D deficiency is associated with an increased risk of PD-related peritonitis, even after adjusting for comorbidities, nutritional status, and physical performance [6]. Interventional studies also demonstrated that oral supplementation of vitamin D may reduce respiratory infection rates [18, 19].

Therefore, we plan to conduct a pilot study to examine the feasibility for a future, full-scale randomized controlled trial (RCT) exploring the effect of oral vitamin D (liquid natural vitamin D3, cholecalciferol; 2000 IU, 0.08 ml) on the risk of PD-related peritonitis in patients who have already experienced an episode of peritonitis. Our hypothesis is that oral supplementation of vitamin D could reduce the risk of subsequent peritonitis.

## Methods

### Trial design and setting

**Fig. 1:**
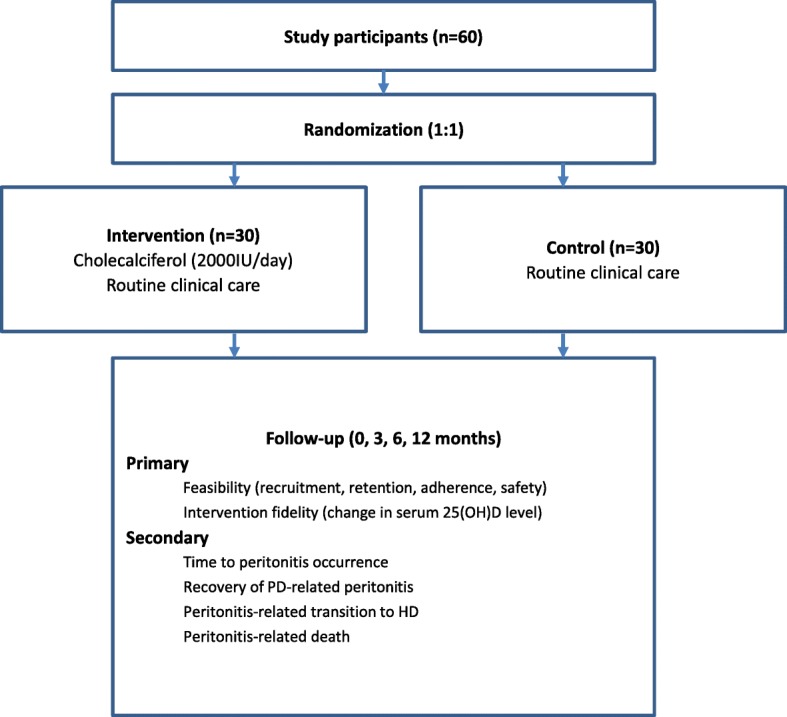
Trial schema. HD hemodialysis, 25(OH)D 25-hydroxyvitamin D, PD peritoneal dialysis

This is a pilot, single-center, randomized, open-label, controlled trial with balanced randomization (1:1) among patients on PD. The protocol of this study was reported adhering to the ‘Standard Protocol Items: Recommendations for Interventional Trials’ statement [20] (Additional file 1). After informed consent, all eligible participants will be randomized to the intervention group with vitamin D supplementation or the control group. Participants will be followed for 12 months. Figure 1 shows the schema of the trial.

The study is being conducted in the PD center of Peking University First Hospital.

### Ethical approval, trial registration, and dissemination

The study was approved by the Peking University First Hospital Research Ethics Committee (Project-ID:2016[1149]), and was registered at Clinicaltrails.gov [21] on 29 August 2017 (NCT03264625 [22]). The results will be disseminated in peer-reviewed journals, and lay summaries will be presented in “ScienceOpen” [23].

### Study population

All patients who have recovered from a PD-related peritonitis at 1 month after the episode will be screened for eligibility. Patients will be recruited if they meet all of the following criteria: aged at least 18 years; undergoing PD for ≥ 1 month and clinically stable; weekly *Kt*/*V* ≥ 1.5 and no clinical uremic symptoms; and serum 25-hydroxyvitamin D (25(OH)D) < 75 nmol/l (30 ng/ml). Patients will be excluded if they have any of the following: received vitamin D2/D3 or drugs known to alter serum 25(OH)D levels during the previous 12 months; have a history of allergic reaction to cholecalciferol; have current or past malignant disease, active hepatitis or hepatic failure, acute systemic infection, active autoimmune diseases, severe digestive malabsorption or eating disorder, or human immunodeficiency virus infection or acquired immune deficiency syndrome (HIV/AIDS); have a high probability (assessed by the recruiting physician) of receiving a kidney transplant or transition to hemodialysis (HD) or dropout due to socioeconomic causes within 6 months; or women who were pregnant or lactating.

Written informed consent will be required from all study participants before their involvement in the trial. Participants can choose to withdraw from the study at any time without affecting their future treatment. Reasons for non-inclusion and withdrawal will be recorded in detail.

### Randomization and blinding

All consenting patients will be registered in the trial and randomized 1:1 to either the vitamin D intervention group or the control group by a computer-generated random number list. An independent medical staff member is responsible for participant enrollment and allocation assignment. The sample size for this pilot study will be 30 patients for both groups (Fig. 1). Due to the nature of the medication intervention, neither participants nor researchers can be blinded. However, both the primary outcome (the assessment of feasibility and fidelity) and the key secondary outcome (time to peritonitis occurrence, recovery of peritonitis, peritonitis-related transition to hemodialysis, and peritonitis-related death) will be evaluated by independent medical staff who are blinded to participants’ allocations. In addition, all laboratory parameters for all participants will be measured by laboratory staff who are also blinded to treatment assignment.

The primary outcome is the assessment of feasibility (recruitment success, retention, adherence, safety) and fidelity (change in serum 25(OH)D level during follow-up) for a large, powered randomized controlled trial to determine the effects of vitamin D on the risk of PD-related peritonitis in the future. Secondary outcomes include time to peritonitis occurrence, recovery of peritonitis, peritonitis-related transition to hemodialysis, and peritonitis-related death (defined as death within 30 days of peritonitis onset).

### Intervention

Participants in the intervention group will receive additional vitamin D (liquid natural vitamin D3, cholecalciferol; 2000 IU) in a dose of 0.08 ml orally per day for 12 months following randomization. In order to assess medication adherence, participants will be asked to take the study medication that is left over for weighing at each clinical visit.

### Control

Participants in the control group will not receive any study drug and will be asked to not take any form of vitamin D supplementation.

### Concomitant treatment

For both the intervention and control groups, all dialysis and other medication prescriptions will be in accordance with routine clinical care and International Society for Peritoneal Dialysis (ISPD) guideline recommendations.

### Baseline evaluation and follow-up

Data collection for this study is composed of two phases: baseline evaluation and follow-up. All of the data will be recorded by the responsible physician on a uniform case report form (CRF). Detailed information collected in each phase is described in the following. Table 1 presents an overview of the data collection schedule during baseline evaluation and follow-up. All participant information will be collected and maintained by trained medical staff to protect confidentiality.

**Table 1:** Data collection schedule

		Baseline	Week 4	Week 8	Week 12	Week 16	Week 20	Week 24	Week 28	Week 32	Week 36	Week 40	Week 44	Week 48
Demographic data, PMH, and medications		X												
Clinical symptoms and adverse events		X	X	X	X	X	X	X	X	X	X	X	X	X
Biochemical indices	Blood routine	X			X			X			X			X
	Blood biochemistry	X			X			X			X			X
	Serum 25(OH)D	X			X			X			X			X
Dialysis adequacy	Weekly *Kt*/*V*	X			X			X			X			X
	Weekly CCr	X			X			X			X			X
Adherence	24-h recall	X	X	X	X	X	X	X	X	X	X	X	X	X

*CCr* creatinine clearance rate, Kt/V urea clearance index, *25(OH)D* 25-hydroxyvitamin D, *PMH* past medical history

#### Baseline evaluation

For all eligible and consenting patients, information regarding demographic data (age, gender, body mass index (BMI), education, marital status, occupation, income, health insurance), dialysis duration, primary disease, comorbidities, current medication, and history of PD-related peritonitis will be recorded. Baseline biochemistry indices (including blood routine, serum creatinine, albumin, alanine transaminase, lipids, electrolytes, parathyroid hormone, and serum 25(OH)D levels), dialysis adequacy (including the urea clearance index (*Kt*/*V*) and creatinine clearance rate (CCr)), and inflammatory biomarkers (interleukin-6, plasminogen activator inhibitor 1) will be evaluated. After randomization, the assigned treatment should be recorded in detail.

#### Follow-up and outcome evaluation

All participants are planned to be followed-up for 12 months, with clinic visits every month. During the follow-up, clinical information including self-reported symptoms and physical examination will be gathered. Biochemistry indices (including blood routine, serum creatinine, albumin, alanine transaminase, lipids, electrolytes, parathyroid hormone, and serum 25(OH)D levels) and dialysis adequacy will be evaluated every 3 months. Participants will be evaluated for compliance by weighing residual liquid vitamin D3 every month. All outcomes and adverse events will be recorded. Please refer to the following sections for detailed outcomes and adverse events.

The primary outcome is assessment of feasibility (recruitment success, retention of participants for 12 months, patient adherence, and safety) and fidelity (change in serum 25(OH)D level between baseline and 12 months). Secondary outcomes include time to peritonitis occurrence, recovery of peritonitis, peritonitis-related transition to HD, and peritonitis-related death (defined as death within 30 days of peritonitis onset). Death not associated with peritonitis, transition to HD not associated with peritonitis, and receiving a kidney transplant will be recorded as competing outcomes.

Recruitment will be determined as the proportion (percentage) of potentially eligible patients recruited into the trial: the numerator would be the number of patients recruited in the trial; the denominator would be the number of patients diagnosed with PD-related related peritonitis during the recruitment period. Successful recruitment will be defined as greater than 40%, the 95% CI (confidence interval) for this cutoff point is 28–52%. Reasons for non-participation from those who were diagnosed with peritonitis but were not recruited will be recorded. Retention will be evaluated by measuring attrition during the follow-up at each clinic visit. The retention rate will be calculated as the percentage of patients who completed the trial among patients recruited in the trial. Successful retention will be defined as > 80% at the end of the 12-month follow-up period. Adherence to taking vitamin D will be evaluated by weighing the vitamin D that is left over at each clinical visit. The adherence rate will be calculated as the percentage of patients who are adherent to the dosing regimen among patients recruited in the intervention group. Participants taking an average dose over 70% of the required dose are considered adherent to the dosing regimen. Acceptable adherence will be defined as greater than 70%. Fidelity will be measured by the difference of change in the serum 25(OH)D level over time between the interventional group and the control group.

For the assessment of safety, both severe and non-severe prespecified adverse events during the study course, including allergic reaction to vitamin D, hypercalcemia, and hyperphosphatemia, will be recorded. Severe adverse events include an AE which fulfils at least one of the following: life-threatening, requires hospitalization, results in disability or congenital abnormality, or requires medical intervention to prevent one of the aforementioned outcomes.

The recovery of peritonitis was defined as complete resolution of peritonitis (normalization of body temperature, resolution of abdominal pain, clearing of dialysate, and PD effluent white blood cell count < 100 cells/μl with < 50% polymorphonuclear cells using antibiotics alone without relapse within 4 weeks of completion of therapy).

### Biochemical testing methods

Blood samples will be analyzed at Peking University First Hospital, Beijing. Biochemical profiles will be tested using an automatic Hitachi chemistry analyzer. Serum 25(OH)D levels will be measured using an electrochemiluminescence method (E601; Roche Diagnostics, Germany).

### Statistical analysis

Statistical analysis will be conducted using SPSS software (version 22.0; SPSS Inc.) and SAS software (version 9.4; SAS Institute). Continuous variables will be expressed as the mean ± standard deviation, median with interquartile range, and percentage. Student’s *t* test, the Mann–Whitney *U* test, and the chi-square test will be used to compare differences in baseline characteristics between the intervention and control groups.

The recruitment rate, retention rate, and adherence rate will be reported as percentages and associated 95% CI. Adverse events will be recorded as the event rate per patient-year. Linear mixed-effects regression models will be used to analyze the change in serum 25(OH)D levels between the two groups. Competing risks Cox regression models will be used to compare the time to peritonitis between the two groups. Logistic regression models will be applied to compare the short-term outcome of subsequent peritonitis.

For all statistical analysis, the level of significance will be set at 0.05.

## Discussion

The present pilot RCT aims to evaluate the feasibility of oral vitamin D supplementation among PD patients and to explore its effects on the risk of PD-related peritonitis. Few interventional studies have been done exploring the effects of vitamin D on preventing infection, especially among PD patients. Therefore, obtaining data on the evaluation of feasibility is the priority in this study. The findings of this pilot study will be used to inform the design and methodology of a definitive study evaluating the efficacy and safety of vitamin D supplementation in the prevention of PD-related peritonitis among patients on PD.

In the general population, there are many RCTs exploring the effects of vitamin D in preventing infections, such as respiratory infections in vitamin D-deficient patients with chronic obstructive pulmonary disease [19], and in children [18]. However, no such study has been undertaken in patients with end-stage kidney disease even though vitamin D deficiency has been shown to be independently associated with PD-related peritonitis [6].

Our study has applied a rigorous design in choosing the time point for patient enrollment. According to previous cohort studies, patients having experienced a peritonitis episode tend to develop a subsequent one after a median time interval of 8 months [5]. Therefore, we focused our attention on the prevention of a subsequent peritonitis episode during a 12-month period among PD patients who have recently recovered from peritonitis, given that this is expected to provide an enriched cohort of patients with a high peritonitis event rate. Based on current data, more than 20% of participants have developed one or more subsequent episodes of peritonitis during a 1-year observation period. As ISPD guidelines recommend a standardized treatment period of 2–3 weeks for PD-related peritonitis [24], we chose to enroll patients into the present study 1 month after the onset of a PD-related peritonitis, as antibiotic treatment would be expected to be completed by this time.

We have adopted a study design using vitamin D and blank control for included patients with vitamin D deficiency. The study design is similar to most previously published randomized controlled trials of vitamin D among the general population [19, 25], CKD patients [26], and the dialysis population [27]. Although recent KDIGO guidelines suggest that vitamin D deficiency be corrected, the level of recommendation is 2C, and whether natural vitamin D supplementation could improve clinical outcomes remains a question that needs further exploration [28], which justifies the ethics of our study.

According to the US Dietary Guidelines, the recommended daily dose of vitamin D for older adults is 600–800 IU per day [29], while a meta-analysis of randomized controlled trials in the general population showed a usual dose of 300–2000 IU per day [30]. In non-dialysis CKD patients, a systemic review indicated that at least 2000 IU vitamin D per day is necessary for ameliorating vitamin D deficiency [31]. In dialysis patients, a randomized controlled trial among maintenance hemodialysis patients showed that a weekly dose of 10,333 IU cholecalciferol for 15 weeks could raise vitamin D levels from 13.3 ng/ml to 23.6 ng/ml [32]. Participants in all of these studies showed an improvement in serum 25(OH)D level, with low incidences of hypercalcemia and hyperphosphatemia. As participants in our study need to take vitamin D for a whole year, after considering the safety, efficacy, and feasibility, we chose to set the dose of vitamin D at 2000 IU. The liquid form of vitamin D was chosen for the sake of convenience in oral administration and evaluation of compliance. According to data from preliminary results of the present study, oral supplementation of vitamin D 2000 IU per day could raise serum 25(OH)D to adequate levels. Serum levels of 25(OH)D increase significantly once oral supplementation of vitamin D is started, reaches a plateau around 40 ng/ml by the 6th month, and thereafter remains stable.

The underlying mechanism for the observed association between vitamin D deficiency and infection in previous studies [6, 15–17] may lie in the role of vitamin D in potentiation of the innate immune system. The immunoregulatory effect of 1,25(OH)_2_D is mediated through the vitamin D receptor, which is present in most immune cells. Vitamin D would upregulate macrophage differentiation, induce the production of antimicrobial peptide cathelicidin, as well as decrease the release of cytokines. Thus, higher levels of vitamin D may result in an increased anti-infectious ability and limit the potential inflammatory damage [13].

Vitamin D deficiency has been identified as one of the common modifiable risk factors for PD-related peritonitis [33]. Exploring whether supplementation of vitamin D could lower the risk of subsequent peritonitis would help identification of a potentially novel and cheap strategy for preventing PD-related peritonitis. Furthermore, the role of vitamin D supplementation on outcomes of PD-related peritonitis will also be explored. The result of this pilot study will determine the feasibility of conducting a full-scale RCT, which may help to better inform shared decision-making by patients and clinicians with respect to the role of vitamin D supplementation regarding the prevention and mitigation of PD-related peritonitis.

## Trial status

This is the first edition of the protocol (September 30, 2016) approved by the ethics committee of Peking University First Hospital. The first participant was enrolled on September 30, 2017. Currently, 42 patients have been recruited into the study and the final recruitment target is 60. Recruitment and follow-up are scheduled to continue until January 30, 2021.

## Supplementary information

**Additional file 1.** SPIRIT 2013 Checklist: Recommended items to address in a clinical trial protocol and related documents.

## Data Availability

The datasets generated during the current study are not publicly available due to the issue of individual privacy, but are available from the corresponding author on reasonable request.

## Abbreviations

25(OH)D: 25-Hydroxyvitamin D
ISPD: International Society for Peritoneal Dialysis
IU: International unit
PD: Peritoneal dialysis
PROBE: Prospective, open-label randomized controlled pilot trial with blinded end-points
RCT: Randomized controlled trial
